# The coral symbiont *Candidatus* Aquarickettsia is variably abundant in threatened Caribbean acroporids and transmitted horizontally

**DOI:** 10.1038/s41396-021-01077-8

**Published:** 2021-08-06

**Authors:** Lydia J. Baker, Hannah G. Reich, Sheila A. Kitchen, J. Grace Klinges, Hanna R. Koch, Iliana B. Baums, Erinn M. Muller, Rebecca Vega Thurber

**Affiliations:** 1 Department of Microbiology, Oregon State University, Corvallis, OR USA; 2 Department of Biology, The Pennsylvania State University, University Park, PA USA; 3 Division of Biology and Biological Engineering, California Institute of Science and Technology, Pasadena, CA USA; 4 Coral Restoration Program, Mote Marine Laboratory, Summerland Key, FL USA

**Keywords:** Population genetics, Phylogenetics

## Abstract

The symbiont “*Candidatus* Aquarickettsia rohweri” infects a diversity of aquatic hosts. In the threatened Caribbean coral, *Acropora cervicornis*, *Aquarickettsia* proliferates in response to increased nutrient exposure, resulting in suppressed growth and increased disease susceptibility and mortality of coral. This study evaluated the extent, as well as the ecology and evolution of *Aquarickettsia* infecting threatened corals, *Ac. cervicornis*, and *Ac. palmata* and their hybrid (“*Ac. prolifera*”). *Aquarickettsia* was found in all acroporids, with coral host and geographic location impacting the infection magnitude. Phylogenomic and genome-wide single-nucleotide variant analysis of *Aquarickettsia* found phylogenetic clustering by geographic region, not by coral taxon. Analysis of *Aquarickettsia* fixation indices suggests multiple sequential infections of the same coral colony are unlikely. Furthermore, relative to other Rickettsiales species, *Aquarickettsia* is undergoing positive selection, with Florida populations experiencing greater positive selection relative to other Caribbean locations. This may be due in part to *Aquarickettsia* proliferating in response to greater nutrient stress in Florida, as indicated by greater in situ replication rates in these corals. *Aquarickettsia* was not found to significantly codiversify with either the coral animal or the coral’s algal symbiont (*Symbiodinium* “*fitti*”). Quantitative PCR analysis showed that gametes, larvae, recruits, and juveniles from susceptible, captive-reared coral genets were not infected with *Aquarickettsia*. Thus, horizontal transmission of *Aquarickettsia* via coral mucocytes or an unidentified host is more likely. The prevalence of *Aquarickettsia* in *Ac. cervicornis* and its high abundance in the Florida coral population suggests that coral disease mitigation efforts focus on preventing early infection via horizontal transmission.

## Introduction

The alpha-proteobacterium “*Ca*. Aquarickettsia rohweri” is a recently discovered bacterial symbiont of many aquatic hosts from around the world [1]. This symbiosis, e.g., a persistent association between two organisms, occurs in reef-building corals (scleractinians), as well as other cnidarians, sponges, and ctenophores [1]. Although fairly ubiquitous, *A. rohweri* may have a more pervasive interaction with Caribbean acroporid corals, as Rickettsiales-like organisms, likely to be *A. rohweri*, have been found in all histological examinations of these coral species since 1975 [2–5]. Among the Caribbean acroporid coral taxa, the highest concentrations of *A. rohweri* are observed in the coral *Acropora cervicornis* following prolonged nutrient enrichment, and resulting in reduced growth of the hosts [6]. In fact, genets of *Acropora cervicornis* that are more susceptible to outbreaks of white band disease (WBD) were recently shown to contain significantly higher abundances of *A. rohweri* [7]. Thus, it is suspected that *A. rohweri* facilitates either the onset or progression of WBD [6, 8, 9], a disease that has contributed significantly to the decline of the reef-building corals, *Ac. cervicornis* and *Ac. palmata* [10, 11]. These two coral species are now so rare that they have been listed as threatened under the US Endangered Species Act and are a major target for restoration efforts [12, 13]. Thus, interrogating *A. rohweri’s*, evolution, transmission route, and roles in initiating and/or mediating disease events may be critical for successful reef management.

Aspects of this bacterial parasite’s biology that are unknown, but are key to understanding its effects on host population dynamics, are how the parasite moves between coral colonies and whether it can move between different coral species. Transmission mode is a major determinant of symbiont population structure and evolution [14–16]. Symbionts that are transmitted vertically, which is from parent to offspring, commonly have limited functional capacities and a reduced genome as they coevolve with and become more dependent on their host [17]. The genome of *A. rohweri* associated with Caribbean acroporids is significantly reduced (1.28 Mbp) and has limited metabolic capacities, including the inability to produce multiple amino acids and ATP [1]. Thus, *A. rohweri* is likely an obligate symbiont dependent on a host for nutrition and energy, and therefore may be transmitted vertically, similar to other closely related and obligate Rickettsiales species, including *Wolbachia* [15, 18].

In symbiotic systems where transmission cannot be observed directly, transmission can be deduced by comparing symbiont and host phylogenies. If the reduced genetic capacity of *A. rohweri* is indeed indicative of vertical transmission by either the coral or the coral’s mutualistic, intracellular algae (Family Symbiodiniaceae), the host and *A. rohweri* phylogenies would be congruent [16, 19, 20]. Conversely, the absence of significant congruence with either the coral or algal symbiont would indicate that *A. rohweri* is likely transmitted horizontally, i.e., through an alternative host or through the environment [21, 22]. *A. rohweri* associates with evolutionarily distant hosts [1], which is comparable to arthropod or plant mediated horizontal transmission of terrestrial Rickettsiales [23, 24]. Although secondary hosts have not been identified yet, possible modes of transmission in the Caribbean include the gastropod *Coralliophila abbreviata* [25], zooplankton [26], or other coral associates. In fact, previously conducted fluorescence in situ microscopy of infected coral polyps did not resolve whether *A. rohweri* is associated with the acroporid coral cells or the coral’s obligate mutualist, *Symbiodinium* “*fitti*.” Rickettsiales-like organisms were observed in the actinopharynx, cnidoglandular bands, gastrodermal mucocytes, oral disk, and tentacles of a healthy *Ac. cervicornis* [8], which are spaces shared with the algal mutualist. All Caribbean acroporid species take up their algal mutualists from the environment upon larval settlement [27], and thus algal mutualists, and perhaps *A. rohweri* with them, are horizontally transmitted. However, Caribbean acroporids mainly propagate through asexual fragmentation [28], resulting in an additional albeit clonal dispersal mode for both the algal symbionts and *A. rohweri*.

Regardless of transmission mode, *A. rohweri* populations may also be structured by coral host species and the environment, although the latter is difficult to disentangle, as one likely co-varies with the other [29]. For example, the location where the first genome of *A. rohweri* was characterized, the Florida Keys, has been exposed to increasing anthropogenic inputs [30, 31] and wide-spread bleaching events [32, 33]. Corals in this area have also experienced multi-year epizootics, including stony coral tissue loss, WBD, and white pox disease [11, 34, 35]. Differential exposure to these stressors may result in dissimilar disease resistance by location, with higher occurrences of disease resistance in Florida (27%) relative to similar populations found in Panama (6%) and USVI (8%) [36]. This in turn may influence the prevalence of infection of the nutrient-stress responsive *A. rohweri* [6, 37]. Although disentangling the impact of host and environment will require further sampling and experimental efforts, our comparative analysis of *A. rohweri* populations provides insight into how infection by this parasitic bacterium may be influenced by the different environmental conditions of each sampling location.

Though *A. rohweri* is capable of infecting a variety of marine phyla, the present study focused on infection of *Acropora* coral found in the Caribbean: *Ac. cervicornis*, *Ac. palmata*, and their hybrid, commonly referred to as “*Ac. prolifera*.” Our objective was to provide an in-depth analysis of *A. rohweri* population structure and acroporid coral infections in the Caribbean. We utilized genomic characterization of the host taxa and their intercellular algal mutualist, *Symbiodinium* “*fitti*,” [29, 38, 39] to investigate possible strain-specific interactions between *A. rohweri* and members of the holobiont. In addition, we studied the diversity of *A. rohweri* infecting acroporids across the Caribbean to understand how quickly this parasite has evolved in this ecosystem [40–42]. Finally, we compared *A. rohweri* genomes to determine the degree of connectivity between populations and the likelihood of reinfection within and between sampling locations, as well as the parasite’s possible mode of transmission.

## Methods

### Sample acquisition and sequencing

Caribbean Acropora samples used in this study were taken from previously published sequence data, and were previously used to characterize *A. rohweri* [1], the cnidarian host [38], and algal symbiont [29] (accession numbers listed in Supplementary Table S1). The sample originally used to describe the bacterial *A. rohweri* reference genome “Acer44” (accession #GCA 003953955) was collected from the Florida Keys as described in Shaver et al. [6], and extracted and sequenced as described in Klinges et al. [1, 6]. Using the reference genome “Acer44,” the presence of *A. rohweri* was evaluated for all samples previously described in Kitchen et al. [38] and Reich et al. [29] (76 samples total; PRJNA473816). These adult coral tissue samples were collected from 12 reefs across the Caribbean, for a total of 23 *Ac. cervicornis*, 30 *Ac. palmata*, and 23 "*Ac. prolifera"*. Samples included in this study were collected between 2001 and 2017 (Supplementary Table S1) and were extracted and sequenced as described in Kitchen et al. [39] and Reich et al. [29, 38]. The methods used to evaluate these samples for *A. rohweri* infection, ecology, and evolution are presented below and summarized in Supplementary Fig. S1.

### Identifying samples infected with *A. rohweri*

Sequences were filtered using bbduk version 36.20 to ensure sequences had a minimum length of 30 and a minimum average quality of 10 [43, 44]. Reads were aligned to the bacterial reference genome *A. rohweri* Acer44 (accession #GCA 003953955), using Bowtie 2 v2.4.1 with the maximum penalty decreased to 4 [45] to quantify the proportion of reads that matched the reference sequence and identify candidates for assembly. Confounding factors to the analysis, such as certain coral hosts or locations resulting in a higher number of reads, or if the date or year sampled impacted the proportion of reads that were identified as *A. rohweri*, were evaluated using one-way analysis of variance (ANOVA). The reads identified as *A. rohweri* were normalized to the total coral host reads as an intrinsic measure of microbial load [46–48] to evaluate the possible impact of coral host and location using two-way ANOVA and post-hoc analysis using Tukey honestly significant difference (HDS) post hoc test within R version 3.6.2 [49]. The impact of reef identity on the proportion of reads identified as *A. rohweri* was also evaluated as a nested factor using one-way ANOVA and Tukey HDS post hoc test.

All samples with greater than 10,000 reads matching the Acer44 genome were de novo assembled using SPAdes version 3.13.1 with the single-cell option to account for possible PCR bias in assembly [50]. Other methods used to construct genomes included (1) removing coral and algal (*Ac. digitifera*: GCF_000222465.1; and *S.* “*fitti*”*:* PRJNA473816) sequences (2) assembling from reads that matched the reference genome and (3) using different binning protocols. However, these alternative methods resulted in genomes that were shorter, lower quality, or more contaminated (data not shown). Thus, we only report data for the SPAdes de novo assembly.

Contigs containing *A. rohweri* genes from Acer44 were identified using BLAST v2.11.0 [51] and binned as potential genomes; any contigs ≤200 bp were excluded from analysis. Of the 19 assemblies, 13 resulted in genomes that were predicted to be greater than 80% complete with less than 2% contamination as evaluated using CheckM [52, 53]. All 14 metagenome-assembled genomes (MAGs) were compared to one another to establish if there were additional species using pairwise average nucleotide identity (ANI) analysis using OrthoANI [52]. Coverage was evaluated using bbmap [43, 44]. All *A. rohweri* MAGs were annotated using Prokka v1.14.6 [54] and orthogroups common to all samples were identified using orthofinder v2.3.9 [55, 56]. A total of 1528 orthogroups were used to identify the pangenome as well as possible host and location-specific orthogroups; these data were plotted using the R-program upsetR [49]. Genes identified as being location-specific were further evaluated using the BLASTX search of the NCBI nr database [51] and the HmmerWeb v2.41.1 using the UniprotKB database [57, 58]; both searches were limited to Rickettsiales.

### Evaluating population structure and evolution of *A. rohweri*

Thirteen *A. rohweri* genomes generated in this study plus the reference Acer44 taken from nine reefs across the Caribbean were used to evaluate potential differences in gene evolution. Phylogenomic trees were constructed with other well-characterized parasitic Rickettsiales species serving as outgroups (Supplementary Table S2). Rickettsiales species were selected on the criterium as being closely related to *A. rohweri* and having multiple completed strains with low percent contamination; this analysis was performed both to characterize *A. rohweri* evolution and to identify possible shared functions. Rickettsiales genomes were annotated using Prokka v1.14.6 and orthogroups were identified using orthofinder v2.3.9 [55, 56]. A total of 143 single-copy orthogroups were common to all samples. DNA sequences of orthologous genes were used to generate a phylogenomic tree. This tree was used as the input for codiversification analysis and identified the root during the construction of single-nucleotide polymorphisms (SNPs) phylogenies. The model parameters used to construct the tree were also used as input for the evolutionary analysis of dN/dS. The DNA sequence of each individual orthologous gene was aligned using MAFFT version v7.453 [59]. Genes were concatenated by sample, and a tree was constructed using IQ-TREE v2.0.3 [60, 61] with 1000 bootstrap replicates. Within IQ-Tree, J-modelTest determined the most likely model was the general time reversible model with empirical base and codon frequencies, allowing for a proportion of invariable sites, and a discrete Gamma model with default four rate categories (GTR+F+I+G4).

Whole-genome phylogenetic trends were also evaluated by identifying SNPs relative to the bacterial reference genome *A. rohweri* Acer44. SNPs common to two or more samples that could be used to construct a SNPs phylogeny were found using the haploid SNP-caller, snippy 4.0-dev2 [62], which implements bwa mem and freebayes to identify high-quality SNPs and assemble a core genome alignment. IQ-Tree was used to construct a tree from core-SNPs with 1000 bootstrap replicates; the model selected was Jukes Cantor and ascertainment bias correction (JC+ASC). SNPs impacting annotated portions of the genome were annotated using SnpEFF version 4.3t to find the likely effects of variants on gene function.

Population structure and strain evolution were characterized using the intrasample nucleotide diversity metric (π) and the fixation index (F_st_). Intrasample nucleotide diversity was also estimated to compare polymorphism in *A. rohweri* populations across locations and host taxa. F_st_ is a measure of genetic population differentiation, ranging between 0 and 1. When two populations are compared and the resulting F_st_ is closer to zero, this implies that populations are freely exchanging alleles. Likewise, if two populations are compared and the resulting F_st_ is closer to 1, then these populations are genetically isolated. To estimate F_st_ and π, SNPs were found by aligning sample reads to the prokka annotated reference bacterial genome Acer44 using bwa mem v0.7.17-r1188, with samclip v0.2 processing allowing a maximum of a 10 clip length and the removal of duplicate alignments using samtools markdup v1.10. To avoid biases that affect variant detection, data were first subsampled to the lowest number of reads (5 × 10^4^) before realigning and then subsampled by the lowest median coverage found in a sample (4x), as suggested by the normalizing protocol outlined in Romero Picazo et al. [22]. Variants were identified using lofreq with a minimum coverage of 10, a strand multiple testing correlation of *p* > 0.001, and a minimum SNP quality of 70. The F_st_ was calculated using the R packages seqinr [22, 63]; this yielded 10–112 SNPs impacting functional genes (Supplementary Table S3). Scripts used for F_st_ and π analysis are publicly available on github repositories (https://github.com/deropi/BathyBrooksiSymbionts). Pairwise comparisons of F_st_ values found in different locations and host taxa were evaluated using one-way ANOVA and Tukey HSD.

Individual orthologous proteins were aligned using MAFFT, and codon alignments were generated using pal2nal [64]. Codon alignments were evaluated by codeml in paml v3.15 [65] using a pairwise comparison, with all other parameters set to approximate the tree-building protocol described above. The result of this analysis was compared to the default parameters for codeml. Because the default parameters were less likely than our model parameters, only the results of our model parameters are presented. Only genes with a dN less than 0, dS between 0.1 and 2, and dN/dS less than 10 were used to find the average dN/dS. This is because pairwise comparisons where dS is less than 0.01 or dN is equal to 0 are similar enough to be considered identical and dS greater than 2 are indicative that synonymous substitutions are near saturation. Similarly, values of dN/dS greater than 10 are considered largely artifactual [66]. From the single-copy orthogroups identified by orthofinder, 9% of the total pairwise comparisons are suitable for dN/dS analysis. Within-species and location comparisons were evaluated using two-way ANOVA and post-hoc analysis using Tukey HDS.

### Evaluating the impact of environment on *A. rohweri* replication rate

Replication rate can be estimated for draft-quality genomes using the index of replication, iRep. Replication is estimated using an algorithm that assumes bi-directional replication from a single origin and accounts for the total change in coverage in genome fragments from reads aligned to a reference genome. A population where all cells are actively replicating would have an iRep of 2, and a population where only a quarter of the cells were replicating would have an iRep of 1.25 [67]. All samples had adequate coverage to perform iRep analysis, but lacked one or more of the criteria to produce a filtered iRep (Supplementary Table S4); the unfiltered result is presented as the genomes constructed are nearly identical (>99% ANI) and do not necessitate the filtering step to account for strain variation and/or integrated phage that can result in highly variable coverage [67]. Differences in the unfiltered rate of replication as a result of host or location sampled were evaluated using two-way ANOVA, and the impact of location was evaluated as a nested factor using one-way ANOVA. All were evaluated using Tukey HDS.

### Identifying likelihood of codiversification of *A. rohweri* with coral or algal symbiont

Both gene-based and whole mitochondrial genome trees were constructed for both hosts and *A. rohweri*. Coral phylogenies were constructed using the mitochondrial control region [68], and full mitochondrial genomes were used to identify the parentage of the F1 hybrid coral “*Acropora prolifera*.” The mitochondrial genomes for each coral host were assembled using two approaches. In the first approach, filtered and trimmed short-read sequences were mapped to the *Ac. digitifera* mitochondrial genome sequence (NCBI: NC_022830.1 [69]) using Bowtie 2 v2.3.4.1 [45] with the parameters for the sensitive mode. Reads were extracted using bamtofastq in bedtools v2.26.0 [70] and then assembled using SPAdes v3.10.1 [71] with various k-mer sizes (-k 21, 33, 55, 77, and 99). In the second approach, we used the de novo organelle genome assembler NOVOplasty [72]. The coral *A. digitifera* mitochondrial genome was used as the seed sequence to extract similar sequences from the original, unfiltered reads for each coral host. Consensus sequences of the mitochondrial genomes for each coral host were created after manual alignment of the sequences from the two approaches using MEGAX [73]. The consensus sequences were run through the web server MITOS [74] to predict genes, tRNAs and rRNAs, and non-coding regions. The phylogenetic relationship of the mitochondrial genomes was inferred with the Maximum Likelihood method using RAxML v8.2.12 [75]. We included eight acroporid mitochondrial genomes from the Indo-Pacific as outgroups [76, 77]. Because the mitochondrial genome can undergo different models of evolution among sites, we ran the genome alignment through PartitionFinder v2.1.1 [78] to determine the best partitioning scheme and substitution models using the greedy algorithm with estimated branches set as linked. In the ML analyses, we used the GTRGAMMA substitution model for the nine partitions. The tree topology with the highest-likelihood based on AIC criterion was reconstructed from 200 replicate trees and nodal support was taken from 1000 bootstrap replicates.

Two phylogenetic trees were constructed for *Symbiodinium* “*fitti*;” one was a gene tree constructed using the marker genes described in Pochon et al. [79], and another was constructed using whole-genome SNPs as described in Reich et al. [29], which excluded any coral samples that had multiple symbiont colonizations. Genes in Pochon et al. [79] (coB, coI, cp23S, nr28S, psbA; [79]) were identified using a BLAST search of the aforementioned de novo Spades assemblies and gene trees were aligned using MAFFT. The *S.* “*fitti*” SNP tree was constructed using 6813 SNPs with quality scores of >200 and that were recovered in all samples from [29]. Raw sequence data for *S.* “*fitti*”-acroporid metagenomes are publicly available on NCBI under SRA project PRJNA473816 [29]. Phylogenies for concatenated gene trees and SNPs trees were constructed using IQ-Tree with 1000 bootstrap replicates; genes and model parameters are described in Supplementary Table S5.

Bacterial and eukaryotic phylogenies were evaluated for significant codiversification using the Procrustes Approach to Cophylogeny (PACo), which uses ultrametric rooted trees to create cophenetic matrices that are evaluated for codivergence 10^5^ times in R [80]. This global fit method evaluates phylogenies that are not fully resolved to evaluate if there is significant codiversification between a host and symbiont species. To evaluate transmission by the coral host, bacterial phylogenomic and SNPs trees were compared to coral phylogenies (whole-genome SNPs trees and mitochondrial SNPs and genes). To evaluate transmission with a *Symbiodinium* host, bacterial phylogenomic and SNPs phylogenies were compared to *Symbiodinium* gene and SNPs trees. Only comparisons with *p* < 0.05 are presented along with the residual sum of squares (*m*^2^) to provide a context for how well the data fit the codiversification model. Significant codiversification analysis was evaluated using a jackknife sum of squares to find those members contributing to the significant association, as they will have values below the 95% residual sum of squares.

### Evaluating vertical transmission using susceptible coral genets

A few days preceding the annual *Acropora cervicornis* spawning events in 2019 and 2020, sexually mature, adult colonies of coral genets “13” and “50” were brought into Mote Marine Laboratory’s Elizabeth Moore International Center for Coral Reef Research & Restoration from their offshore coral nursery in the lower Florida Keys [7, 36]. Genets were originally collected from nearby reefs (<20 km maximum linear distance) and had been growing in the nursery for at least 5 years (Muller et al. [36]; Supp File 5). The small spatial scale over which genets were originally sourced suggests that these belong to the same population (Baums et al. 2010; Drury et al. 2016). On land, colonies belonging to the two genets were isolated from each other. Spawning, fertilization, settlement, and grow-out were conducted following published protocols and under standardized conditions [81–89]. The coral *Ac. cervicornis* is a simultaneous hermaphrodite, and broadcast spawning activity was observed in August during the predicted peak spawning window for this species [90]. Gamete bundles of eggs and sperm were collected from each genet. After bundle dissolution, sperm was isolated from the eggs by filtration using a 100 µm mesh sieve. Triplicate subsamples were concentrated via low speed centrifugation and then snap-frozen and stored at –80 °C. Triplicate subsamples of 50–100 eggs per genet were also snap-frozen and stored at –80 °C. The remaining egg stock from genet 13 and sperm stock from genet 50 were combined and allowed to undergo fertilization for one hour (cross “13e × 50s”) using an optimal sperm concentration of approximately 10^6^ sperm cells/mL [81, 84]. The lack of viable offspring produced for the reciprocal cross in both years suggests some degree of incompatibility between eggs of genet 50 and sperm of genet 13, a phenomenon previously observed between other acroporid genets [83]. Embryos were reared in replicate cultures with filtered seawater at room temperature (27 °C). Approximately 1 week later, triplicate subsamples of 50 larvae were snap-frozen and stored at –80 °C. The remaining larvae were settled in 5-gallon glass tanks using unconditioned ceramic settlement substrates and live, crushed up crustose coralline algae as the settlement cue. After settlement, sexual recruits were reared in a common garden at Mote’s land-based coral nursery. Recruits and juvenile corals were maintained in flow-through mesocosms (“raceways”) with running seawater, from which algal symbionts were naturally acquired. Fouling algae was mitigated using intertidal herbivorous snails (*Batillaria* spp.). Daily husbandry consisted of raceway siphoning and basting of the substrates to remove snail detritus. Flow rates were maintained between 4 and 6 L/min.

Samples were collected at the gamete (egg and sperm), larval (<1 week of age), recruit (~2 months), and juvenile (~1 year) stages from a 13e × 50s cross. A total of 50 larvae were sampled and pooled, 15 individual sexual recruits were sampled and pooled together, and 6 polyps were sampled and pooled from 5 individual juveniles. Six samples were selected for further analysis and preserved in liquid nitrogen: (1) egg and (2) sperm stock from genet 50, (3) egg stock from genet 13, (4) larvae from the 2020 13e × 50s cross, (5) recruits from the 2020 13e × 50s cross, (6) juveniles from the 2019 13e × 50s cross. A seventh sample (also of juveniles from the 2019 13e × 50s cross) was preserved in DNA/RNA shield to assess the effect of preservation method on *A. rohweri* quantification. DNA was extracted from early life stage coral samples from both preservation methods using the Omega EZNA DNA/RNA Isolation Kit (Omega, Norcross, GA, USA). Extracted nucleic acids were stored at –80 °C until further processing. Quantitative polymerase chain reaction (qPCR) was performed on the seven early life stage samples (each in triplicate) using primers designed to target the *Acropora cervicornis* actin gene (as an endogenous control) and an *Aquarickettsia*-specific gene, ATP/ADP translocase *tlc1*, using iQ 10 μL SYBR Green Supermix (Bio-Rad, Hercules, CA, USA). An 149 bp section of the *tlc1* gene of *A. rohweri* was amplified using 0.3 μM sequence-specific primers (F: 5’-GGCACCTATTGTAGTTGCGG-3’, R: 5’-CATCAGCTGCTGCCTTACCT-3’), and the actin gene of *Acropora cervicornis* was amplified as in Wright et al. [91] as an endogenous control [91]. A sample of *Acropora hyacinthus* (collected from Mo’orea, French Polynesia in 2017) was used as a calibrator, as this species expresses actin but lacks *A. rohweri* (unpublished data). A positive control (a sample of adult coral genet 50 with known quantity of *A. rohweri*) and a no-template control (molecular grade water) were prepared using the same methods and quantified simultaneously. A 35-cycle qPCR was performed on an Applied Biosystems 7500 Fast Real-Time PCR System (Applied Biosystems, Foster City, CA, USA) at the OSU Center for Genome Research and Biotechnology, using cycling parameters selected to minimize mispriming: An initial denaturation step of 3 min at 95 °C, followed by 35 cycles of 95 °C for 15 s, 56 °C for 30 s, and 72 °C for 30 s. Melt curve analysis was performed to identify any off-target products and relative quantification methods were used to assess expression of tlc1. Relative quantification determines the change in expression of the target sequence (tlc1) in tested samples relative to the same sequence in the calibrator sample (*Ac. hyacinthus*). Differences in expression between samples were analyzed using one-way ANOVA. Results were confirmed through non-quantitative PCR of the *tlc1* and 16S rRNA genes (515F‐806R primer set [92]) using AccuStart™ II PCR ToughMix (QuantaBio, Beverly, MA, USA) and subsequent gel electrophoresis on a 1.5% agarose gel with Invitrogen 100 bp DNA Ladder (ThermoFisher Scientific, Waltham, MA, USA).

## Results

### MAGs generated from multiple locations suggest that *A. rohweri* associates with many Caribbean Acropora but is more abundant in *Acropora cervicornis*

In order to evaluate the population dynamics of *Ca*. Aquarickettsia rohweri with its acroporid coral hosts, we identified bacterial sequences in previously published genome studies [1, 29, 38]. Using a series of bioinformatic steps (Supplementary Fig. S1), we found *A. rohweri* sequences in all *Acropora* specimen collected from across the Caribbean as part of the Kitchen et al. [38, 39] and Reich et al. [29] studies, including sites within Belize, the Florida Keys, Curaçao, and the US Virgin Islands (Supplementary Table S1). Before normalization, samples had between 38 and 1,219,071 sequence reads that aligned to the bacterial reference genome, *A. rohweri* Acer44 (GCA 003953955). Sequencing effort was not significantly different for either coral hosts or locations (*p* = 0.67 for coral host; *p* = 0.28 for location). Similarly, sampling time (both date and year) did not impact the proportion of reads identified as *A. rohweri* (*p* = 0.78 for date; *p* = 0.13 for year). The *A. rohweri* identified reads were then normalized to the total number of coral host reads as an intrinsic measure of microbial load [46–48]. Using this method, *A. rohweri* read abundance was found to vary according to coral host and sampling location (Supplementary Fig. S2), but not by the specific reef site where samples were taken within each regional location (Tukey adjusted *p* > 0.5). Reads from *A. rohweri* made up a greater proportion of the total sequences in *Ac. cervicornis* samples relative to the hybrid “*Ac. prolifera*” (on average, 4× more reads, Tukey adjusted *p* = 0.0002) and *Ac. palmata* (86× more reads, *p* < 0.0001) samples. In addition, *A. rohweri* reads made up a greater proportion of the total reads from all samples collected in Belize relative to Curaçao (46× more, *p* = 0.007). Although limited to *Ac. cervicornis* corals, a greater proportion of *A. rohweri* reads were found in Belize samples relative to those collected from Florida (1.6× more, *p* < 0.00001) and the USVI (1.8× more, *p* = 0.017).

### The coral parasite, *A. rohweri*, phylogeny differentiates by location, not by host

To further interrogate the biogeography and population structure of *A. rohweri*, we then assembled the reads and constructed and annotated MAGs from these sequence libraries. Only samples from the corals *Ac. cervicornis* and the hybrid “*Ac. prolifera*” from Florida, USVI, and Belize contained sufficient reads to construct Aquarickettsia MAGs (deposited under PRJNA666461). These MAGs were used to conduct comparative phylogenomics of *A. rohweri* from different locations (Belize: 7, Florida: 4, USVI: 3) and different host taxa (*Ac. cervicornis*: 12, hybrid “*Ac. prolifera*”: 2). Six of the MAGs constructed were less complete than the original *A. rohweri* genome (<98.9% complete), but four from Belize were 100% complete with no contamination and a larger N_50_ value than the reference assembly (Table 1). All *A. rohweri* MAGs were at least 1.21 Mbp and had a >99% ANI in pairwise comparisons to the reference genome and one another (Table 1 and Supplementary Table S6).

**Table 1:**
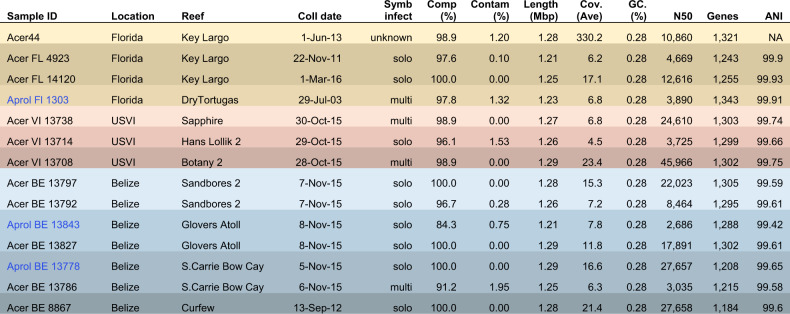
Collection data and genome quality information for *A. rohweri* constructed from PRJNA473816, including the reference genome from Klinges et al. [1].

Coral host taxa include *Ac. cervicornis* (Acer) and “*Ac. prolifera*” (Aprol). *Symbiodinium* “*fitti*” colonization status, whether by a single strain or multiple strains, were evaluated in Reich et al. [29]. Completeness, contamination, length, percent GC, and N50 were found using checkm, coverage was found using bbmap, genes are the total number of prokka annotations, and the ANI to the reference sequence *A. rohweri* Acer44 was found using orthoANI.

**Fig. 1:**
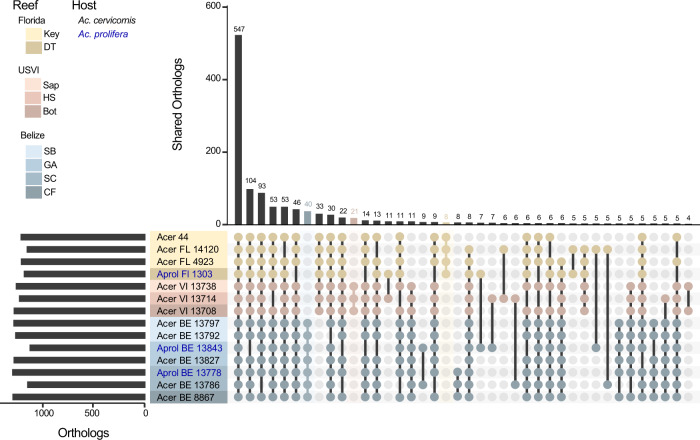
The number of orthologous genes in *A. rohweri* identified using orthofinder for each sample. Location-specific genes are highlighted in the bar plot by location color: Florida (yellow, 8 orthogroups), USVI (pink, 21 orthogroups), and Belize (blue, 40 orthogroups). Host identity is noted in blue text for “*Ac. prolifera*” (Aprol) and black text for *Ac. cervicornis* (Acer), although no orthogroups are exclusive to either host taxon.

From the assembled bacterial genomes, between 1184 and 1343 genes were annotated per sample and 98.4% of the sequences were identified as belonging to 1528 orthogroups (Fig. 1). No orthogroups were exclusive to either coral host. Greater than 30% of these sequences were identified as single-copy orthogroups shared by all samples. Location-specific orthogroups were all single-copy. Florida had 8 unique orthogroups, Belize had 40, and USVI had 21 (Fig. 1). The majority of location-specific genes were annotated by Prokka as hypothetical proteins; however, searching for the function of these genes against the NCBI nr database and the HmmerWeb UniprotKB database resulted in additional annotation, with only about 40% of the genes remaining unidentified (Florida:3, Belize 19, and USVI 8). Functional genes specific to locales include: the protein transfer gene *secA* in Florida, as well as multiple transport genes in Belize and USVI, as well as two genes involved in the type II toxin-antitoxin system in Belize (Supplementary Table S7).

**Fig. 2:**
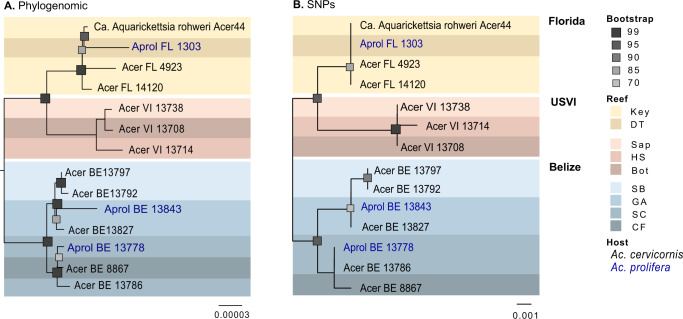
*A. rohweri* phylogenies for all metagenome-assembled genomes (MAGs). **A** Phylogenies generated using orthofinder to identify orthogroups to construct a phylogenomic tree, rooting based on comparison to other Rickettsiales (Supplementary Fig. S3). **B** Phylogeny of core-SNPs (15) generated by snippy and rooted based on the outcome of the orthofinder tree. Bootstrap values greater than 70 are shown.

Phylogenomic analysis of the orthologous genes of the de novo assembled *A. rohweri* MAGs showed variation among samples collected from different locations, irrespective of host. Comparison to well-studied host-associated Rickettsiales species (*Ehrlichia chaffeensis*, *Rickettsia prowazekii*, *Rickettsia rickettsii*, and *Wolbachia pipientis*) resulted in the identification of 71 share orthologous genes. The phylogenetic tree constructed from DNA of these orthogroups resulted in all newly constructed *A. rohweri* genomes tightly clustered near the reference genome, *A. rohweri* Acer44 (Supplementary Fig. S2). Limiting phylogenomic analysis to just *A. rohweri* MAGs resulted in clear differentiation between samples collected across the Caribbean and north-west Atlantic; Belize *A. rohweri* are distinct from those isolated in the Virgin Islands and Florida, regardless of host identity (Fig. 2A). Furthermore, the *A. rohweri* isolated from the hybrid “*Ac. prolifera*” does not form a separate clade from those isolated from *Ac. cervicornis* even when collected from the same location. This is especially evident in Belize samples where *A. rohweri* genomes constructed from the hybrid “*Ac. prolifera*” and *Ac. cervicornis* taken from the same reef were more closely related than *A. rohweri* collected from the hybrid “*Ac. prolifera*” from a neighboring reef. Clustering by location was similar in the SNP analysis (Fig. 2B), despite recovering a surprisingly small number of SNPs per sample (*n* = 11–2345) after filtering (minimum read depth = 10, minimum fraction of 0.9, and a minimum mapping quality of 100; Supplementary Table S8). Relative to the reference genome, Acer44, samples had low levels of genetic polymorphism (0.63 ± 0.69 SNPs/kilobase) (Supplementary Table S8). This resulted in few SNPs that are shared by multiple samples (*n* = 15, i.e., those that are informative in phylogenetic analysis).

### Levels of genetic isolation suggests *A. rohweri* hosts are not being reinfected

**Fig. 3:**
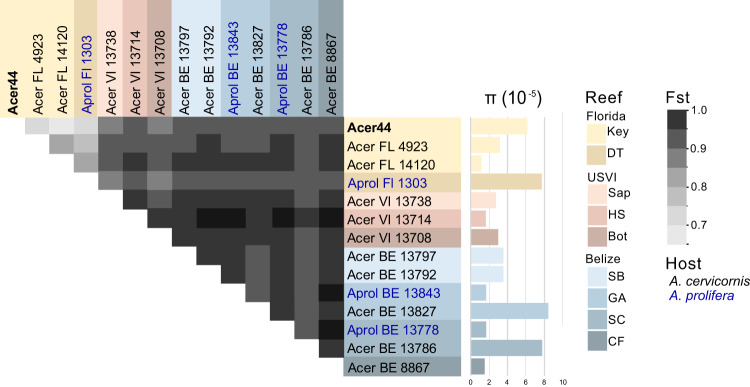
Pairwise comparisons of the fixation indices (F_st_) and intrasample nucleotide diversity (π) for each sample from each location, generated using methods outlined in Romero Picazo et al. [22]. Samples originating from Belize shown in blue, Florida in yellow, and the USVI in orange. All comparisons between USVI and Belize samples resulted in an F_st_ of >0.86, whereas samples with the Florida populations were between 0.64 and 0.83.

Analysis of SNPs within annotated portions of the *A. rohweri* genomes indicates that although samples have similar levels of genetic diversity, the *A. rohweri* sampled from distinct colonies are relatively genetically isolated from one another. This is even true of samples collected from the same reef (Fig. 3). *A. rohweri* intrasample nucleotide diversity (π) did not differ among sampling location or host, and was on average 1.85 ± 0.8 × 10^–5^; this suggests that neither host nor sampling location is leading to higher levels of genetic variation within samples. However, pairwise comparison of intrasample *A. rohweri* SNPs between coral colonies mostly resulted in a F_st_ of 0.86 or greater. This level of genetic isolation among *A. rohweri* infections suggests that *A. rohweri* populations are not mixing among coral hosts once an infection is established. This level of genetic isolation was found in all pairwise comparisons in USVI and Belize, and was even observed in pairwise comparisons of samples taken from the same reef (Sandbores, Glovers Atoll, and South Carrie Bow Cay). Samples from Florida had significantly lower levels of genetic isolation relative to USVI and Belize (F_st_ = 0.65–0.83; Tukey adjusted *p* < 0.0001). Although these values still imply *A. rohweri* cell populations found in Florida coral colonies are somewhat genetically isolated from each other, these data suggest that Florida *A. rohweri* populations are mixing more often than those found in colonies from the USVI or Belize. The aforementioned trends account for *A. rohweri* found in both *Acropora* coral taxa, as host identity did not affect the level of genetic isolation (Tukey adjusted *p* = 0.9).

### Most SNPs are in areas that moderately impacted function, with transposases most impacted

All samples had high-quality, intersample SNPs identified using snippy [62] with annotated functional genes relative to the reference genome *A. rohweri* Acer44. Of those SNPs found in functional genes, a majority of the SNPs (on average 62%) were identified as resulting in new amino acids (missense mutations) (Supplementary Table S9). Transposases were also found to be heavily impacted by mutations that likely have a moderate impact on function, as annotated by SnpEFF (Supplementary Table S9). Three samples from Belize (Aprol BE 13843, Acer BE 13797, and Acer BE 13792) were found to have a nonsense mutation, resulting in a stop codon earlier than would be expected for genes in the IS66 family transposase ISDpr4. Four additional transposases were identified as having mutations resulting in new amino acids in all Belize and USVI samples, including IS6 family transposase ISCca2.

### *A. rohweri* is undergoing positive selection

**Fig. 4:**
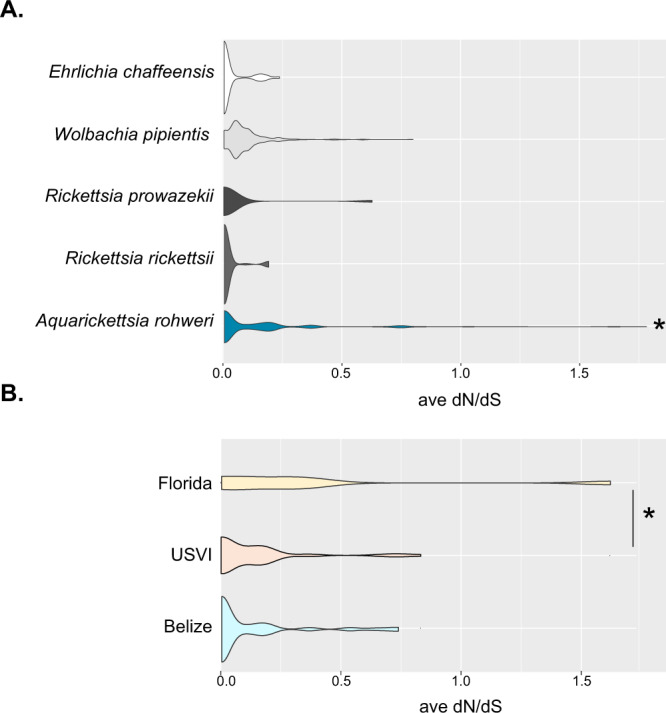
Plots of average dN/dS values for whole-genome comparisons of prokka annotated genes. **A** Average dN/dS of closely related well-studied Rickettsiales species were all lower relative to *A. rohweri* (*p* < 0.0001). **B** Average dN/dS of *A. rohweri* is significantly greater in Florida than USVI (*p* = 0.048).

MAGs from this study were compared to well-studied Rickettsiales relatives of *A. rohweri* to evaluate whether populations of *A. rohweri* are undergoing neutral or positive selection relative to other closely related parasites. This was evaluated using pairwise comparisons of samples using dN/dS, which is the ratio of the number of nonsynonymous substitutions per nonsynonymous sites to the number of synonymous substitutions per synonymous site. Lower values of dN/dS indicate stronger purifying selection, that is selection against deleterious mutations to maintain function [93]. Values of dN/dS approaching 1 indicate positive selection or the selection of new mutations into the population. *A. rohweri* had the highest mean dN/dS, with a much broader distribution of dN/dS values relative to all other Rickettsiales species (36–89% greater dN/dS, *p* < 0.0001) (Fig. 4A and Supplementary Table S10). This indicates that *A. rohweri* is undergoing greater positive selection than closely related Rickettsiales. Comparison of dN/dS of *A. rohweri* across the two *Acropora* coral host taxa ("*Ac. prolifera"* and *Ac. cervicornis*) did not result in significantly different dN/dS (*p* =0 .06; data not shown), but location did affect dN/dS. There was a higher median dN/dS for *A. rohweri* from Florida relative to those from USVI (65% higher, *p* = 0.048) (Fig. 4B). All populations of *A. rohweri*, regardless of location or host, had some genes undergoing positive selection (Supplementary Table S11). Most of these genes were identified by Prokka as hypothetical, meaning that this program predicted that these regions are coding regions, but their function has yet to be identified. Of those that were not hypothetical, GTPase Era, DUF2312 domain-containing protein, and the Bifunctional protein FolD were consistently undergoing some level of positive selection (0.5–0.9 dN/dS) in all samples taken from Belize. The genes experiencing the strongest positive selection (≥1.0 dN/dS) were the 50S ribosomal protein L13, which was found in multiple comparisons of samples from Florida and Belize, and in the type IV secretion system protein VirD4, which was seen in a single comparison between a sample from Florida versus the USVI (Acer FL 4923: Acer VI 13714) (Supplementary Table S11).

### *A. rohweri* from Florida samples exhibit higher replication rates

**Fig. 5:**
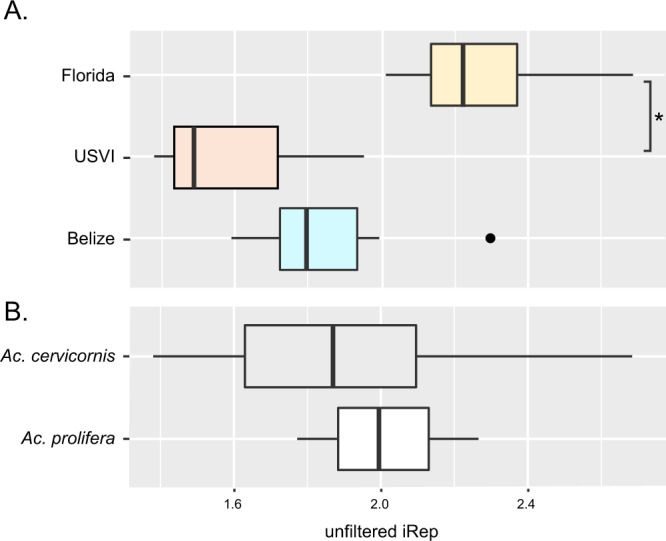
The estimated rate of replication using iREP. The average and range of unfiltered iREP estimates are given for each location (**A**) and coral taxon (**B**); significant differences are noted with an asterisk.

Because previous studies have indicated that *A. rohweri* increases prevalence in response to nutrient exposures, the in situ replication rate is likely to be indicative of areas of sustained exposure to nutrients. MAGs from Florida corals had consistently higher estimated unfiltered rates of replication (iRep); however, this was independent of coral host taxon (Tukey adjusted, *p* = 0.3) (Fig. 5 and Supplementary Table S4). This indicates that *A. rohweri* is not replicating faster in the hybrid “*Ac. prolifera*” vs *Ac. cervicornis*. The unfiltered iRep of samples from Florida was 19% higher than samples from Belize and 30% higher than samples from USVI. A single sample from Belize had as high an iRep as Florida samples (Acer BE 13786); this sample was taken alongside five other Belize samples and also from the same reef as an additional sample (Aprol BE 13778 from South Carrie Bow Cay). Florida samples had a significantly higher estimated rate of replication than those taken from the USVI (*p* = 0.04) but significant differences in the replication rate were not seen when comparing samples collected from the same reef within a region (*p* = 0.44).

### Codiversification analysis and qPCR of coral offspring suggests *A. rohweri* is horizontally transmitted

To evaluate the various possible methods of transmission, multiple codiversification analyses were performed to compare *A. rohweri* evolution with the two common eukaryotic members of the holobiont, *Acropora* and the dinoflagellate symbiont *Symbiodinium* “*fitti*.” This analysis found that neither the host acroporid nor endosymbiotic dinoflagellate (*S.* “*fitti*”) was significantly codiversifying with *A. rohweri*, and thus neither is solely responsible for transmitting the bacteria to the next generation via vertical transmission. All phylogenetic methods used to analyze the coral diversity of samples infected with *A. rohweri* resulted in differentiation between *Ac. cervicornis* and the hybrid “*Ac. prolifera*” (Supplementary Figs. S3 and S4), which was not observed in *A. rohweri* phylogenies (Fig. 2 and Supplementary Fig. S5). Codiversification analysis among *A. rohweri* and coral phylogenies largely were not significant. Although coral phylogenies constructed using the mitochondrial control region identified by Vollmer et al. [68] were significantly codiversifying with *A. rohweri* phylogenomic and SNPs trees (*p* = 0.02), the *m*^2^ values (measure of fit) indicate the data do not fit the codiversification model and are therefore highly unlikely (>800). Coral phylogenies constructed using the whole mitochondrial genome also did not show evidence of codiversification with either *A. rohweri* phylogenomic or SNP phylogenies (*p* < 0.01 and *m*^2^ > 330). This was recapitulated in the jackknife sum of squares of both PACo analysis, which did not identify any samples as significantly contributing to codiversification (Supplementary Table S5).

In addition, comparison of the algal symbiont *S.* “*fitti*” dinoflagellate phylogenies to the bacterial *A. rohweri* phylogenies found that these two members of the holobiont also do not significantly codiversify. The phylogeny constructed using the genes recommended in Pochon et al. [79] resulted in placement of all symbionts in the combined dataset as *Symbiodinium* “*fitti*” (ITS2 type A3, formerly “Clade A”; Supplementary Fig. S6). The gene tree showed a clear differentiation between the samples collected from Florida relative to the USVI and Belize samples; however, USVI and Belize samples formed a single clade, which is dissimilar from the clustering that occurred in either *A. rohweri* phylogeny (Fig. 2). Though the *S.* “*fitti*” gene tree showed some codivergence with *A. rohweri*, the *m*^2^ values indicate the data do not fit the codiversification model and are therefore highly unlikely (*m*^2^ > 600), and jackknife analysis only identified 0–2 samples contributing to this trend (Supplementary Table S5). Phylogenetic analysis using SNPs found that *S.* “*fitti*” genomic variation primarily partitioned to host coral species rather than biogeographic location [29] (Supplementary Fig. S7). The *S.* “*fitti*” SNP and gene trees indicated that there was no significant codivergence between *S.* “*fitti*” and *A. rohweri*. (Supplementary Table S5).

Quantitative PCR was utilized to detect *A. rohweri* in early life stages of *Ac. cervicornis*. Using a primers developed specifically for the *tlc1* gene in *A. rohweri*, qPCR was performed on gametes (egg and sperm), larvae (<1 week of age), recruits (~2 months), and juveniles (~1 year) of an outcross produced between two *Ac. cervicornis* parent genets with microbiomes dominated by *A. rohweri* (genotypes 13 and 50 *sensu* Muller et al. [36], Klinges et al. [7]). While adult *Ac. cervicornis* corals collected from the Mote in situ coral nursery had high expression of *tlc1* relative to the calibrator sample (*Ac. hyacinthus* with no *A. rohweri*), amplification of *tlc1* across all early life stage samples was not significantly different from the calibrator sample and led to the failure of the thresholding algorithm, indicating that *A. rohweri* infection of these samples was unlikely (Supplementary Fig. S8).

## Discussion

*Ca*. Aquarickettsia rohweri infection was found in every sample of acroporid corals taken from across their Caribbean and north-west Atlantic geographic range, with *Ac. cervicornis* corals consistently yielding more *A. rohweri* reads relative to *Ac. palmata* and the hybrid “*Ac. prolifera*” (Supplementary Fig S2A and Supplementary Table S1). Assuming the proportion of *A. rohweri* to host reads are indeed reflective of infection status [46–48], the higher numbers of *A. rohweri* reads in *Ac. cervicornis* may in part explain the increased disease susceptibility of this taxa relative to *Ac. palmata* [93]. The relative resistance of *Ac. palmata* to *A. rohweri* infection may be attributed to environmental factors such as depth, innate host immunity, or defenses mounted by the host microbiome [94–96]. Determining which factors may be leading to resistance in *Ac. palmata* and the hybrid is a valuable area of further research.

The coral *Ac. cervicornis* yielded higher read numbers of *A. rohweri*, but both *Ac. cervicornis* and “*Ac. prolifera*” hosted sufficient reads to construct *A. rohweri* genomes of similar length and quality as the *A. rohweri* reference genome Acer44. Phylogenomic analysis using orthologous genes and SNPs indicate the bacteria infecting Caribbean acroporids are specific to the collection location and not the host taxon (Fig. 2). This is in contrast to the only other acroporid symbiont with population genetic information, the dinoflagellate *Symbiodinium* “*fitti*.” Genomic variation of *S.* “*fitti*” loosely partitions to host taxa, regardless of reef location [29]. These contrasting patterns of population structure indicate that the forces shaping the two coral symbionts are different despite their shared host taxa. Similar differences in the population structures between symbionts co-infecting a shared host include terrestrial symbionts populating aphids and whiteflies [23, 97, 98].

*A. rohweri* populations from the three sampling locations form separate clades in phylogenomic and SNP phylogenies, with Florida and USVI samples likely sharing a closer ancestral lineage than Florida and Belize (Fig. 2). The USVI samples, being sister to the Florida samples, suggest that *A. rohweri* dispersal is not primarily via the Caribbean current that passes from US Virgin Islands through Belize to Florida. A barrier to gene flow has been identified between the eastern and western Caribbean for coral [99, 100]. Similar genetic differentiation by location has been observed for the *Ac. cervicornis* sequences of the same samples used in this analysis [38]. Even though seasonally variable surface currents connect all sampling locations [100, 101], and all samples are genetically similar (relative to the reference genome, all samples were >99% ANI), there was clear differentiation among Florida, the US Virgin Islands, and Belize *A. rohweri* populations (Figs. 1 and 2).

*A. rohweri* collected across the Caribbean have low levels of genetic polymorphism with <2500 SNPs relative to the reference genome of 1.28 Mbp (Supplementary Table S8). Thus, *A. rohweri* may be considered monomorphic [*sensu*, [102–104]]. Lower levels of genetic polymorphism are correlated with virulence in some bacteria, such as pathogens *Yersinia pestis* and *Bacillus anthracis* [102, 104]. However, comparable levels of genetic polymorphisms are found in the bioluminescent mutualists *Ca*. Photodemus katoptron and *Ca*. Photodemus blepaharus [103]. The role of *A. rohweri* in coral disease is an active area of research, and thus it is difficult to interpret how the low levels of genetic polymorphisms in *A. rohweri* influence its parasitic role, but it is notably low for a symbiont spanning such a large geographic range.

Of the subset of SNPs that impact functional regions, most (62%) resulted in a change to the amino acid identity and therefore likely affect protein function (Supplementary Table S9). Although the majority of genes impacted by missense mutations were hypothetical proteins, some gene annotations were identified as transposases. Moreover, the single gene found to have acquired a stop codon in all USVI and Belize samples was within the transposase ISDpr4. The loss of transposases and the eventual loss of these gene regions is characteristic of long-term obligate symbionts [15, 105]; therefore, the *A. rohweri* genome may still be actively reducing through the loss of mobile genetic elements.

Although the *A. rohweri* were closely related to each other and are phylogenetically clustering by sampling location, there were surprisingly high levels of genetic isolation, even within a single reef (Fig. 3). Pairwise comparisons of the fixation indices between all samples indicated extreme genetic isolation between *A. rohweri* populations from distinct coral colonies, indicating that genetic mixing among populations and thus reinfection of coral colonies with *A. rohweri* is unlikely to occur between or even within a reef. Genetically isolated populations of *A. rohweri* may be the result of competitive exclusion once a coral is infected or a consequence of subsequent infections being relatively rare. The most extreme case was observed in comparing samples collected from the same reef in Belize, as all pairwise comparisons had an F_st_ of 0.95 or greater. The lowest level of pairwise genetic differences was observed in Florida, which implies a slightly higher probability of reinfection among coral colonies relative to the other locations, but laboratory studies would be needed to evaluate whether this is due to host or environmental factors. Thus, despite low genetic diversity observed overall, genetic diversity was distributed such that locations and samples were highly differentiated suggesting that *A. rohweri* infection may occur earlier in the coral lifespan and propagate within the host over 30–838 years [106, 107] with little to no genetic mixing among *A. rohweri* populations from distinct coral colonies occurring thereafter. However, at this stage we do not yet know when the parasite establishes infection.

Our work also shows that *A. rohweri* is undergoing greater positive selection relative to closely related parasitic Rickettsiales species, with genes involved in speciating and virulence undergoing the greatest degree of positive selection (Fig. 4A). While the identity of the coral host did not have an impact, sampling location did affect the degree of positive selection (Fig. 4B). The average dN/dS of Florida samples is 2.7 times greater than samples from USVI and 2.8 times greater than those in Belize. Although differences in dN/dS were not observed for all samples at each location, these trends were observed in a subset of the comparisons between all sampling locations. The higher positive selection in Florida populations may be due in part to the higher estimated rates of replication observed in those samples (Fig. 5), but further study would be need to evaluate this trend. Genes that were associated with ribosomal assembly, L13 and GTPase ERA, which assemble 50S and 30S ribosomal proteins, respectively, were undergoing positive selection in a subset of the samples. The consequence of ribosomal-associated genes undergoing positive selection is unknown, but it may be indicative of speciation occurring between the different sampling locations. Another gene undergoing positive selection in a subset of samples across locations was the Type IV secretion system-coupling protein VirD4. VirD is essential to T4SS and is involved in substrate recruitment, which plays a role in oncogenic DNA transfer and virulence in Agrobacterium [108–111]. Thus, positive selection in VirD may be affecting how *A. rohweri* in Florida populations interact with their host species.

Though microscopy found *A. rohweri* living in close proximity to coral cells and S. “*fitti*” [8], neither are likely transmitting the parasite vertically (Supplementary Table S5). Coral larvae seemed the most likely method for transmission across the Caribbean, as larvae can travel long distances as plankton (>500 km) [100]. Similarly, algal symbionts could provide the necessary nutrients to *A. rohweri* and facilitate parasitic infection when *S.* “*fitti*” is acquired by juvenile coral hosts [112–114]. It is also possible that the parasite could be carried alongside either member of the coral holobiont as they reproduce asexually, however, similar to sexual reproduction, this would have resulted in congruence between parasite and host phylogenies and significant codiversification. Yet, codiversification analysis of both SNPs and gene-based phylogenies resulted in neither coral nor *S.* “*fitti*” having clear codivergence with *A. rohweri*. In addition, qPCR evaluation of early life phases (<1 week to 1 year) from disease susceptible coral genotypes known to harbor *A. rohweri* [7] failed to detect the bacteria (Supplementary Fig. S7). The reduced metabolic capabilities of *A. rohweri* [1] and the lack of evidence for a dormancy pathway also suggests that the bacteria is unlikely to survive long periods in the environment as free-living bacteria. However, *A. rohweri* has retained some flagellar genes [1] and flagellum maybe involved in some aspect of transmission or symbiosis. It is therefore most likely that the bacteria are transmitted via an alternative method that would provide the necessary nutrients. One such method may be through the movement of coral mucocytes coupled with some abrasion or inoculation event. In histology studies of *Ac. cervicornis*, Rickettsiales are very commonly found in coral mucocytes that are released into the environment [8]. Mucocytes filled with the parasites would provide a potential source of *A. rohweri* to the surrounding water and available for ingestion by non-infected conspecifics. In addition, coral predation may influence infection, as corallivores such as ciliates, fireworms, or fishes may leave abrasions for entry of the parasite or they may serve as a vector for transmission. Future studies are needed to identify the mode of *A. rohweri* transmission, but the results will further inform Acroporid management efforts in the Caribbean.

Overall, the results of this study show that *A. rohweri* infection differs among coral hosts and locations, is evolving at different rates across its host’s range, and is horizontally transmitted. While this work has revealed the population structure of both a bacterial parasite and its marine host along with new insights into the transmission mechanisms of the parasite, several questions remain unknown and should be investigated in the future. For example, the mode of parasite is transmitted remains unknown. Is mucocyte release and subsequent consumption a primary mechanism, are there secondary vectors, or is there a mobile stage of the parasite yet undiscovered. In addition, we do not know when the transmission of parasite occurs during coral ontogeny. However, these findings do suggest new pathways to the study of *A. rohweri* and its potential contribution to coral diseases in the Caribbean. For example, exploring possible host or microbiome-based deterrents of *A. rohweri* infection of *Ac. palmata* [115] through cross-taxon reinfection or transcriptomic studies may be valuable to the conservation of Caribbean acroporids. In addition, Florida may be a unique focal point for the study of how *A. rohweri* infection impacts coral disease progression. Several *Ac. cervicornis* and a hybrid “*Ac. prolifera*” from the Florida Keys host high concentrations of *A. rohweri* that tend to be less isolated, undergo greater selection in speciation and virulence genes, and are propagating faster than in other sampling locations. Thus, further research into environmental stressors and host responses in this population will be invaluable to our understanding of pathogen evolution, its role in coral disease, and the restoration and recovery of this fragile ecosystem.

## Supplementary information

Supplementary Tables

Supplementary material

## Data Availability

All sequences used are available in the SRA in PRJNA473816 and assembled *A. rohweri* genomes are accessible under PRJNA66646.
