# Supplementary material for: The coral symbiont Candidatus Aquarickettsia is variably abundant in threatened Caribbean acroporids and transmitted horizontally
Source: ISME J. 2021 Aug 6;16(2):400–11. doi: 10.1038/s41396-021-01077-8 (PMC8776821; doi:10.1038/s41396-021-01077-8)
Supplement: Supplementary file 2 — Supplementary material [file 41396_2021_1077_MOESM2_ESM.docx]

Fig. S1: A flow chart of the analysis performed to identify: the number of *Aquarickettsia* reads per sample (dark blue); *Aquarickettsia* orthologs for dN/dS analysis, phylogenomic tree construction, and find location specific genes (green); Fst and pi for population of *Aquarickettsia* (yellow); *Aquarickettsia* SNPs and their impact (orange); *Aquarickettsia* in situ replication rates (grey). Additionally, this diagram shows how these analysis and those of the coral and algal phylogenies fit into Paco codiversification analysis (pink).

Fig. S2: *A. rohweri* read abundance normalized to the total number of coral host reads as an intrinsic measure of microbial load, evaluated as a factor of: (A) coral taxon; (B) sampling location; and (C) coral taxon and sampling location.

Fig. S3: The coral mitochondrial control region generated using genes and outgroups as described in Vollmer et al., 2002, generated using the model HKY+F; shown as the ultrametric tree input used in Paco analysis.

Fig. S4: A phylogeny constructed for the coral mitochondrial genome constructed as described in Kitchen et al., 2019, generated using the GTR+F+G4 model; shown as the ultrametric tree input used in Paco analysis.

Fig. S5: A bacterial phylogenomic tree contracted using well-studied Rickettsiales outgroups listed in Table S2, constructed using the GTR+F+I+G4 model.

Fig. S6: A *Symbiodinium ‘fitti’* phylogeny constructed using the genes recommended in Pochon et al., 2014 including coB, coI, cp23S, nr28S, psbA generated using the GTR+F+G4 model; shown as the ultrametric tree input used in Paco analysis.

Fig. S7: A *Symbiodinium ‘fitti’* phylogeny constructed as outlined in Reich et al., 2019, generated using the GTR+F+G4 model; shown as the ultrametric tree input used in Paco analysis.

Fig S8: Relative quantification (RQ) of early life stage samples, calculated in reference to Acropora hyacinthus calibrator (RQ of 1). An RQ of 3000 means that the expression of the target gene was 3000 times higher in that sample compared to the calibrator. RQ is calculated as 2^-ΔΔCt, where ΔΔCt is the difference in ΔCt between sample and calibrator, and ΔCt is calculated as the difference in Ct value between target gene and endogenous gene (actin). Ct represents the exact point in the PCR protocol (40 cycles) at which amplification was detected. Higher Ct values indicate less of the target gene was present, and more cycles of PCR were necessary to detect the target gene.
